# Dual-imaging model of SQUID biosusceptometry for locating tumors targeted using magnetic nanoparticles

**DOI:** 10.1186/s12951-015-0069-5

**Published:** 2015-02-12

**Authors:** Jen-Jie Chieh, Kai-Wen Huang, Yi-Yan Lee, Wen-Chun Wei

**Affiliations:** Institute of Electro-Optical Science and Technology, National Taiwan Normal University, Taipei, 116 Taiwan; Department of Surgery and Hepatitis Research Center, National Taiwan University Hospital, Taipei, 100 Taiwan; Graduate Institute of Clinical Medicine, National Taiwan University, Taipei, 100 Taiwan

**Keywords:** Magnetic nanoparticle, Tumor, Dual imaging, Scanning superconducting-quantum-interference device

## Abstract

**Background:**

For intraoperative imaging in operating theaters or preoperative imaging in clinics, compact and economic integration rather than large and expensive equipment is required to coregister structural and functional imaging. However, current technologies, such as those integrating optical and gamma cameras or infrared and fluorescence imaging, involve certain drawbacks, including the radioactive biorisks of nuclear medicine indicators and the inconvenience of conducting measurements in dark environments.

**Methods:**

To specifically and magnetically label liver tumors, an anti-alpha-fetoprotein (AFP) reagent was synthesized from biosafe iron oxide magnetic nanoparticles (MNPs) coated with anti-AFP antibody and solved in a phosphate buffered saline solution. In addition, a novel dual-imaging model system integrating an optical camera and magnetic scanning superconducting-quantum-interference device (SQUID) biosusceptometry (SSB) was proposed. The simultaneous coregistration of low-field magnetic images of MNP distributions and optical images of anatomical regions enabled the tumor distribution to be determined easily and in real time. To simulate targeted MNPs within animals, fewer reagents than the injected dose were contained in a microtube as a sample for the phantom test. The phantom test was conducted to examine the system characteristics and the analysis method of dual images. Furthermore, the animal tests were classified into two types, with liver tumors implanted either on the backs or livers of rats. The tumors on the backs were to visually confirm the imaging results of the phantom test, and the tumors on the livers were to simulate real cases in hepatocellular carcinoma people.

**Results:**

A phantom test was conducted using the proposed analysis method; favorable contour agreement was shown between the MNP distribution in optical and magnetic images. Consequently, the positioning and discrimination of liver tumors implanted on the backs and livers of rats were verified by conducting in vivo and ex vivo tests. The results of tissue staining verified the feasibility of using this method to determine the distribution of liver tumors.

**Conclusion:**

The results of this study indicate the clinical potential of using anti-AFP-mediated MNPs and the dual-imaging model SSB for discriminating and locating tumors.

## Background

Tumor imaging is a crucial medical practice that is performed after positive tumor screening results have been confirmed by blood tests. By using medical imaging, clinicians can analyze the phase and distribution of tumors to determine treatment options such as surgery. Medical imaging procedures are classified into structural or functional imaging. Structural imaging is developed on the basis of the physical characteristics of the tissues, whereas functional imaging is developed on the basis of the biological features of tumors or characteristics of bioprobe-mediated nanoparticles, which is an image contrast medium. Hence, simultaneous positioning and discrimination of tumors can be achieved by incorporating structural imaging and functional imaging. For example, structural imaging methods, such as computed tomography (CT) [1] or magnetic resonance imaging (MRI) [2], can be combined with functional imaging methods, such as radioactive positron emission tomography (PET). However, for biosafety, bioprobe-mediated magnetic nanoparticles (MNPs) without radioactive risks are increasingly adopted as a contrast medium in functional imaging [3] for MRI.

In addition, sensitive imaging methodologies such as MRI, CT, or PET are generally difficult to implement in both general medical clinics and clinical practices, particularly because the equipment is expensive to install and maintain. For example, using MRI devices is restricted to preoperative diagnosis because of the high cost of constructing a shielding room with few metal objects, and also the expense involved in maintaining liquid helium coolants. Hence, for the intraoperative positioning of tumors, alternate navigation surgery methodologies involve using robotic or hand-held probes in integrated systems composed of a gamma camera and ultrasound [4] or an optical video [5].

To conform to the requirements of the diverse imaging technologies used in various clinical practices such as preoperative diagnosis and intraoperative positioning of tumors, various functional contrast media have been developed as multimodal contrast media, such as MNPs coated with radioactive or fluorescent [6] indicators in addition to bioprobes [7]. However, using such complex contrast media is costly, and using multimodal nanoparticles involves high biorisk. Hence, simple contrast mediums with biosafety and economic viability, such as gadolinium and iron-oxide MNPs without coated radioactive or fluorescent materials, are approved by the USA Food and Drug Administration [8,9]. According to a previous study, iron-oxide MNPs are superior to gadolinium because gadolinium MNPs induce kidney disease [10]. An imaging technique for intraoperative positioning of tumors must therefore be developed to expand the application of iron-oxide MNPs beyond MRI preoperative diagnosis.

Recently, developments for noninvasively examining MNP distributions have been focused on the magnetic characteristics of MNPs. For example, to examine the remanent properties of MNPs, a static superconducting-quantum-interference device (SQUID) sensor or a pickup coil in a shielded sensing unit detects the magnetized and moved sample from distant magnetization coils or magnets [11,12]. This method is suitable for in vitro tests because of the fast sample movement. The biomedical application of SQUID relaxometry based on MNP relaxation [13-15] is limited to in vitro or ex vivo tests because in addition to the requirement of the shielding environment, the involved signals are sensitive to the dynamic and size distribution, hydrodynamic size, and temperature of MNPs in animals. Furthermore, magnetic particle imaging technologies based on the nonlinear characteristics of MNPs under high field gradients can be used to image the three-dimensional distribution of MNPs. However, the practical application of such technologies is limited because they are difficult to integrate with other real-time structural imaging methods (e.g., MRI) [16].

Conversely, because of the alternate-current (AC) susceptibility of MNPs under low magnetic fields, scanning SQUID biosusceptometry (SSB) examinations of dynamic MNP distributions in the liver, heart, and tumors of animals for pharmacokinetics [17], metabolism [18], and tumor targeting [19] have yielded satisfactory agreement with other biological examinations. The earliest SSB technologies enabled magnetic functional measurement, but an optical video camera was not incorporated. SSB is advantageous because it does not require a shielded environment, animal torsos can be scanned easily because the device can be handled similarly to an ultrasound probe, and liquid nitrogen involves low maintenance costs. Moreover, the AC susceptibility of the MNPs employed in this study has certain advantages. The AC excitation field contributed the centrifugal force to bioprobe-coating MNPs; nontargeted molecules were then removed. Based on this unique characteristic, immunomagnetoreduction (IMR) assays required no complex and washing steps in in vitro blood tests, unlike the enzyme-linked immunosorbent assay currently used in clinics [20,21]. Similarly, the utility of the centrifugal force in in vivo tests, such as in this study, could improve the targeting specificity of bioprobe-coating MNPs for the correctness of tumor label. Furthermore, AC-susceptibility technology can be operated in an unshielded environment by using a general lock-in amplifier, which is superior in comparison with remanent- or relaxation-based technologies that require an expensive shielded environment.

This study proposed a novel SSB method that integrates an optical video camera into the vertical pickup coils, which is in contrast to the planar pickup coils of early SSB technologies. In addition, the integrated and compact probe can scan arbitrarily along an animal torso in an unshielded environment as an ultrasound probe. Novel SSB methods can be used to immediately fuse magnetic and optical images. Thus, SSB is a powerful tool for preoperative diagnosis and the intraoperative positioning of tumors. Although the spatial resolutions of 2-dimensional SSB fusion images are less accurate than those of three-dimensional MRI images for preoperative diagnosis, using 2-dimensional SSB fusion images is adequate for tumor positioning and safer than using gamma cameras or fluorescence imaging for intraoperative positioning. To verify the feasibility of using the proposed SSB technique for surgical navigation, simple anti-alpha-fetoprotein (AFP)-mediated MNPs were employed to conduct both phantom and animal tests, in which AFP was used as a biomarker in rats with tumors of the liver.

## Results and discussion

### Phantom test

**Figure 1:**
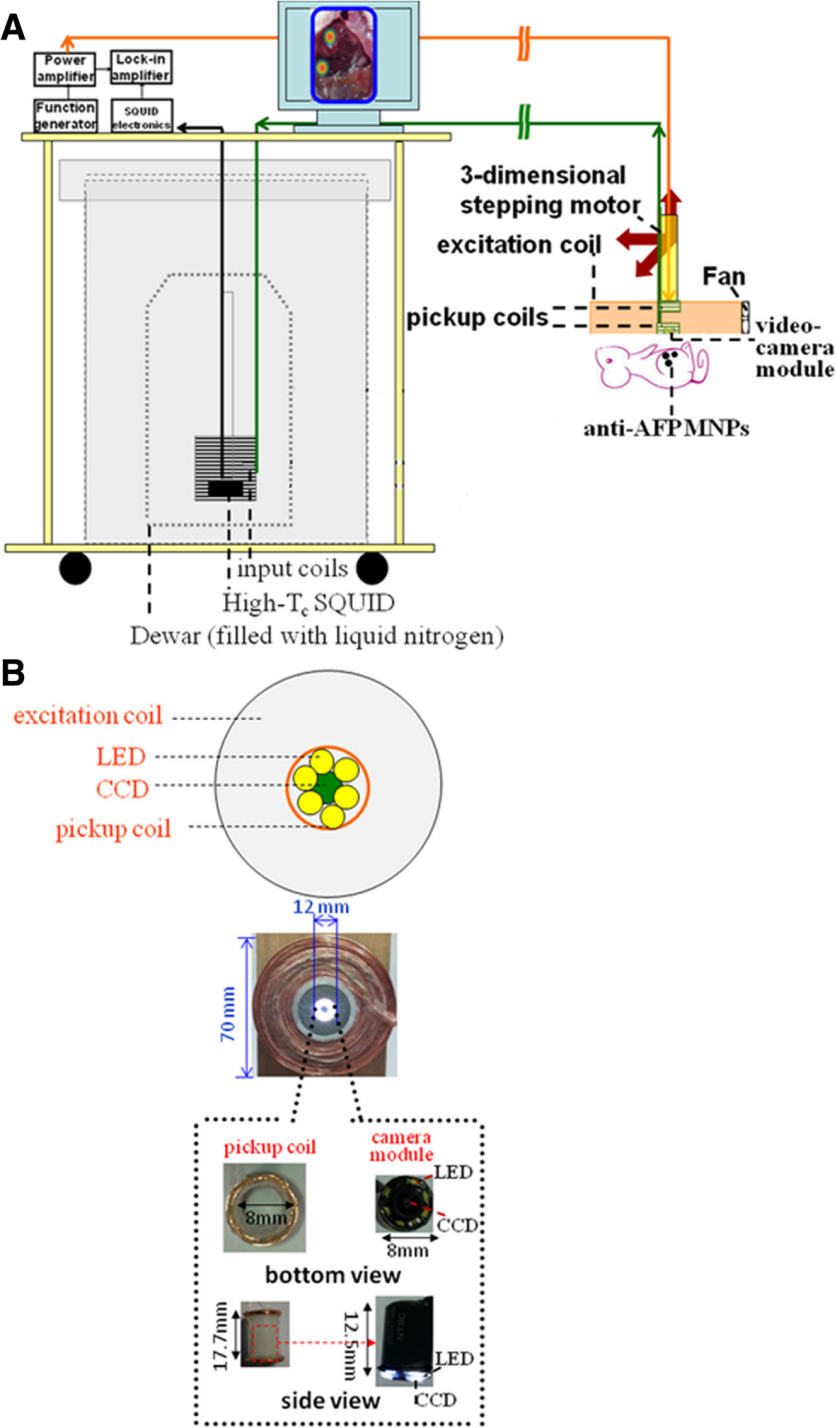
**The dual-model SSB developed for simultaneous optical imaging and magnetic imaging.** Scheme for the examination of tumor rats **(A)**. Bottom view of the scanning probe **(B)**.

**Figure 2:**
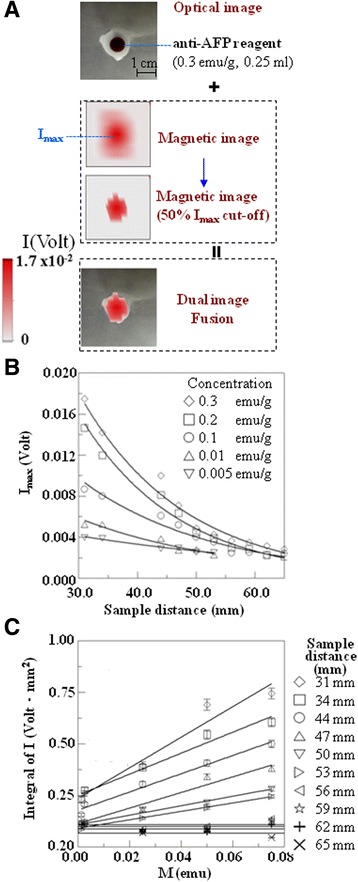
**The phantom test.** Microtest tubes were filled with anti-AFP reagents with concentrations of 0.3, 0.2, 0.1, 0.01, and 0.005 emu/g that were 0.25 g in weight. The image process for the optical image, the magnetic images composed of I and only I larger than 50%, and the fused image **(A)**. The dependence of I_max_ of the red spot in the magnetic image on the sample distance and the sample concentration **(B)**. The dependence of the integral of I on the sample distance and the sample magnetism **(C)**.

The dual-imaging model SSB shown in Figure 1 was used to capture optical and magnetic images of microtest tubes filled with the anti-AFP reagents. The injection dose for animals in this study was 0.9 g with anti-AFP reagents at a concentration of 0.3 emu/g. Figure 2A shows dual-images of the microtest tube filled with 0.3 emu/g and 0.25 g, which is approximately 28% of the injection dose, as well as the overlaid images. In Figure 2A, the optical image shows a top-down view of one of the microtest tubes, as well as magnetic images of the same test tube. In the magnetic images, the upper red spot indicates the raw signal intensity I, whereas the lower magnetic image indicates filtered signals where I is higher than 50% of the peak signal intensity I_max_ in each red spot, not in the entire image. In other words, the upper magnetic spot has more signals with I lower than 50% of I_max_ compared with the lower one.

The magnetic signals were distributed spherically from the sample; therefore, the red spot in the raw magnetic image covers a larger area than that indicated by the optical image. However, the red spot in the filtered magnetic image is similar to the distribution shown in the optical image. The spatial contour error between the red spot in the filtered magnetic image and the brown circle in the optical image is within 3 mm. Hence, the red spot in the filtered magnetic image indicates the MNP distribution shown in the optical image.

Consequently, the sensitivity of this system was studied by using the maximum detectable distance of the same phantom sample. The line graph in Figure 2B shows that the I_max_ of the red spots in the magnetic image decreased as the sample distance between the top surface of the anti-AFP reagents and the bottom surface of the scanning probe unit was increased from 31 to 66 mm. Furthermore, I_max_ decreased as the reagent concentration was reduced from 0.3 to 0.005 emu/g, with Fe concentration ranging from 1.95 mg/g to 32.5 μg/g. This shows that the detectable distance was approximately 56 mm for the samples with a concentration of 0.1–0.3 emu/g, whereas that for the samples with a concentration of 0.005–0.01 emu/g was approximately 44 mm, which is sufficient for detecting MNPs in tumors implanted in the livers or grafted onto the backs of animals [22,23]. In other words, the sensitivity to the amount of Fe in the MNPs was approximately 250 μg at 56 mm and 12.5 μg at 44 mm. Although these discussed distances between the sample and sensing probe were tens of millimeters in this study, and the measurement was performed in an unshielded environment, the minimal detection of MNPs was tens of pictograms at the short distance of 2 mm; this is similar to the results reported by previous research [12]. Furthermore, the detectable distance can be increased for clinical applications involving humans by simply modifying the equipment, such as by increasing the product of the area and current of the cylindrical excitation coil, rather than by injecting more MNPs, which would increase toxicity. For example, the expected detection distance can be increased to approximately 15 cm, which is approximately the half thickness of a general belly, if this product increases tens of times; this reason is the excitation field increases with this product while the sample distance is much larger than the radius of excitation coil based on Biot-Savart law. In addition, if the imaged tumor is large, the detection limit improves because of increased target MNPs.

Figure 2C shows that the integral of I, which represents the product of the sum of I values greater than 50% of I_max_ and the pixel spacing, decreased with the distance between the sample and the scanning probe, but increased with the sample magnetism, which is the product of the reagent concentration and weight. The minimal value was observed at approximately 0.05 Volt.mm^2^, which can be considered as the sensitivity of the integral of I. Moreover, evaluating the level of magnetism in the sample (i.e., MNP amount) was feasible by using the integral of I as a reference.

### Animal test of exterior liver tumors

**Figure 3:**
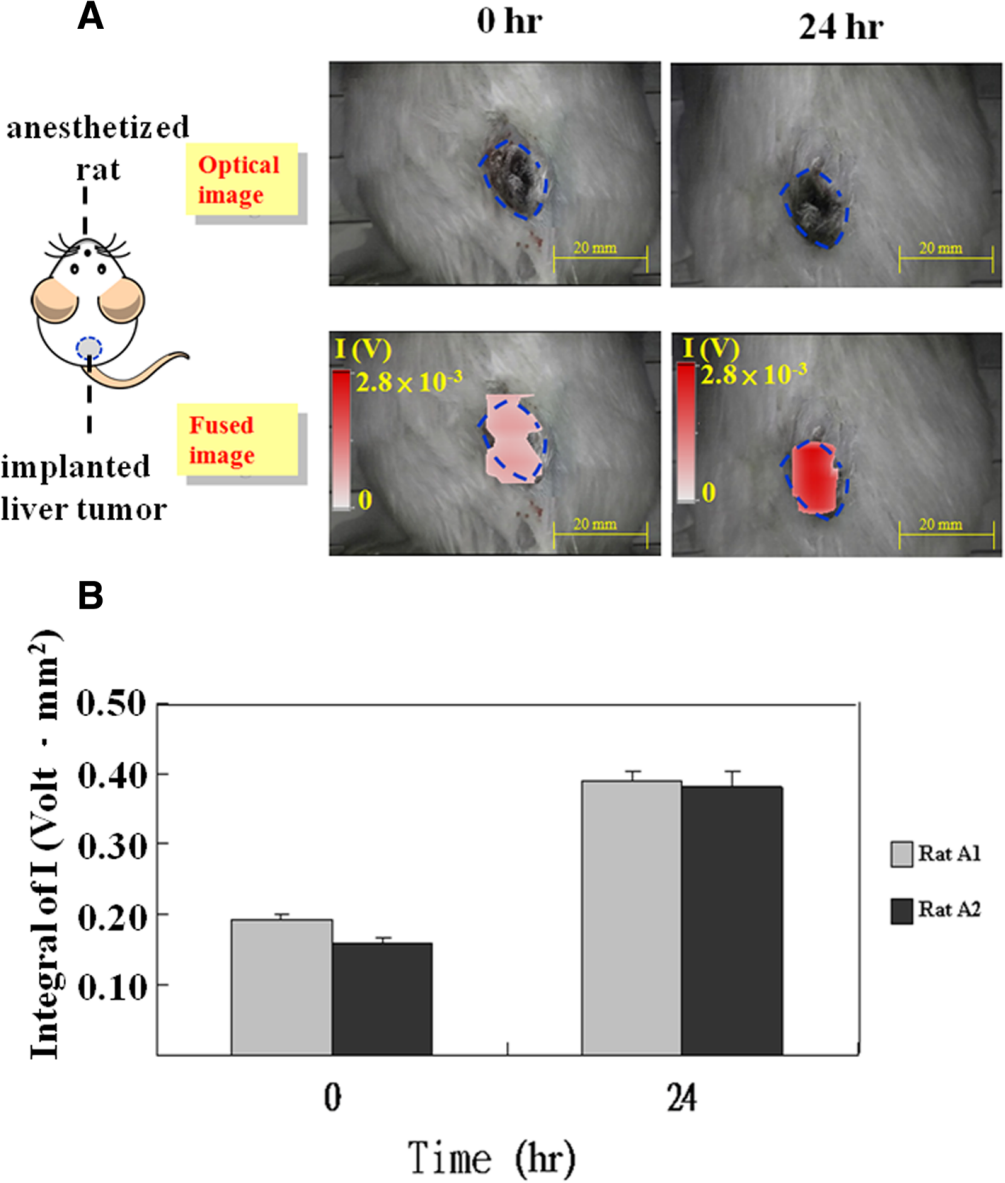
**The animal test for the rats with a liver tumor on the back.** The optical image and the fused image at 0 h and 24 h for 1 tumor rat **(A)**. The average sum of I at 0 and 24 h for 2 tumor rats **(B)**.

Figure 3 shows the results of locating a tumor in the back of one of the rats. The figure shows that at 0 h, before injection of the MNPs, the integral of I was low, but it was higher than the background level because of the presence of weakly paramagnetic materials in the tissue, such as red blood cells. The tumor region generally expressed high I values because it was close to the scanning probe, causing it to dominate the integral of I at 0 h. At 24 h, most of the I of the red spot were high because of the high accumulation of anti-AFP MNPs on the back tumors [19]. Moreover, by 24 h, the anti-AFP MNPs were biodegraded by organs in the mononuclear phagocyte system, which is also called the reticuloendothelial system or macrophage system, and then excreted through other organs [18]. Therefore, at 24 h, the red spot was distributed in the back tumors only. Furthermore, the I of one tumor rat (Figure 3A) and the sum of the I of 2 tumor rats (Figure 3B) were apparently larger at 24 h than at 0 h (ie, before injection). However, the repeatability errors at 24 h and at 0 h were relatively small.

In the optical and fused images in Figure 3A, the blue dotted line and the red spot represents the contour of the implanted tumors and the distribution of anti-AFP MNPs, respectively. The blue dotted line and red spot exhibit adequate consistency, with a spatial error of 5 mm at both 0 h and 24 h. Thus, the SSB can be employed to locate MNP-targeted tumors. Furthermore, these results verify that the integral of I is a suitable indicator for detecting anti-AFP MNPs in tumors when the dual-imaging model SSB is used for in vivo imaging. Hence, the fused images revealed the spatial and functional results of the MNP-targeted tumors, and this targeting phenomenon is in agreement with a similar study that used an identical dose of the same anti-AFP reagent [23].

### Animal test of interior liver tumors

**Figure 4:**
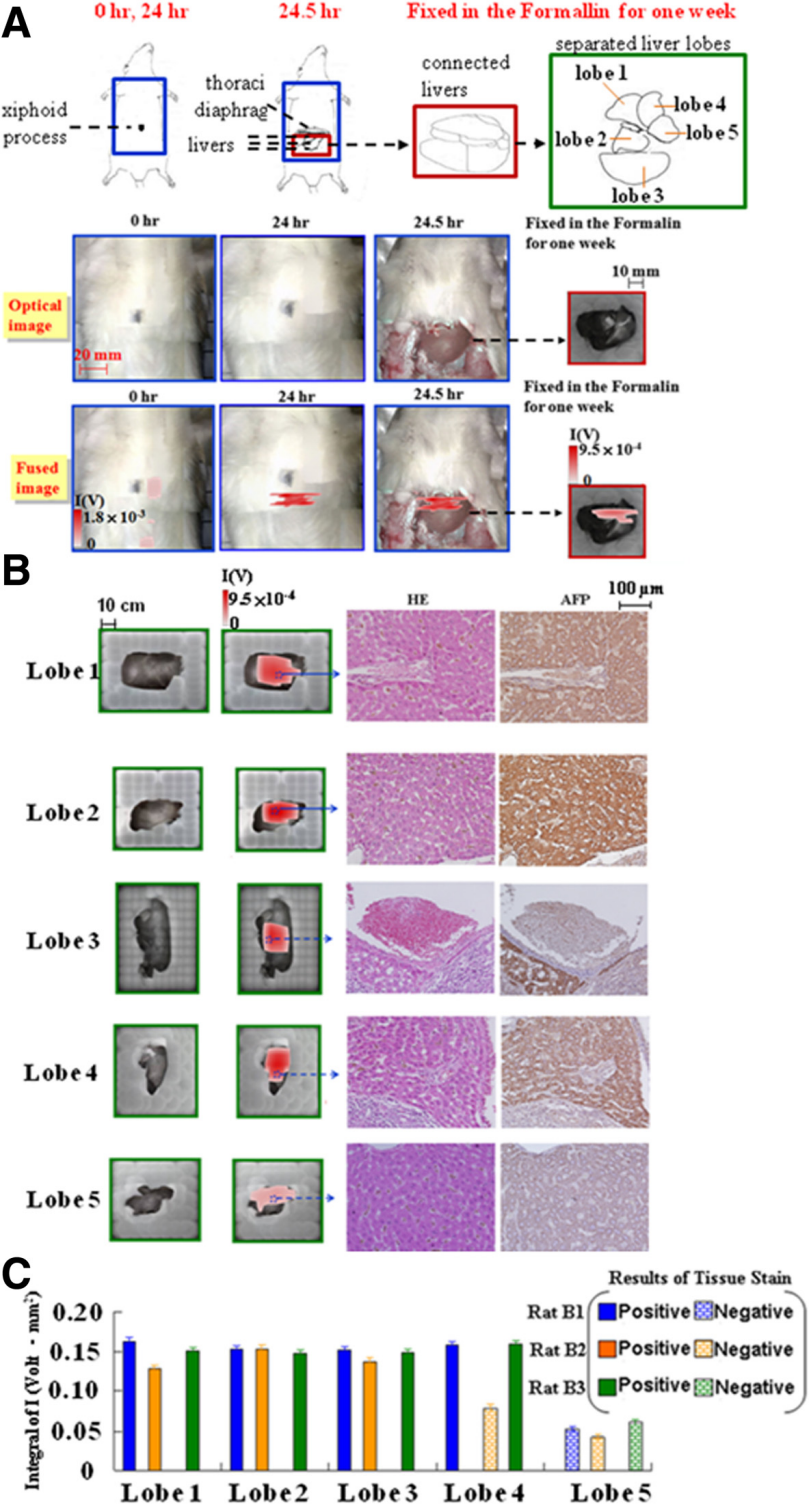
**The animal test for 3 rats with a liver tumor in the liver. Rat B1** was imaged in a supine position at 0 h and 24 h, and with its belly opened at 24.5 h. Its liver was immersed in diluted formalin with 10% concentration for 1 wk. Both the stacked block and separated lobe livers were magnetically examined ex vivo **(A)**. After identifying the red-spot and no-spot regions in the separated lobe livers by using the dual-model SSB for **Rat B1**, one small piece of liver tissue in the red-spot region was confirmed by HE and AFP staining **(B)**. The comparison between the integral of I according to the dual-model SSB and the positive and negative judgment according to the tissue staining for separated liver lobes (Lobes 1–5) of 3 tumor rats **(Rats B1–3)** **(C)**.

The results of the animal tests for the surgical navigation of the liver tumors within the liver lobes are depicted in Figure 4. In the fused images of Rat B1’s unexposed abdomen (Figure 4A), few red spots were observed at 0 h; however, the red spots were clearly observable in the liver region at 24 h. Consequently, exposing the abdomen for in vivo imaging at 24.5 h revealed nearly identical red spots, indicating that the skin did not interfere with the magnetic measurement.

Furthermore, after immersing the livers in formalin for 1 week (wk), the red spot on the preseparation liver lobes in the ex vivo image at 24.5 h was similar to that of the livers in the in vivo image of the exposed livers at 24.5 h, except in the lower left region (Figure 4A). This difference was probably because the short sample distance yielded a strong I in the in vivo test, whereas the lower-left part of the stacked livers was highly sustained by the other organs in the body. Conversely, the long distance yielded a weak I in the ex vivo test, and the lower-left part of the stacked livers was fixed without support from other organs. Similarly, the I of the ex vivo test was weaker because of the long sample distances for some regions of the stacked livers without the support of other organs. In other words, the distance between the scanning probe unit and each liver lobe was shorter in the in vivo test than in the ex vivo test. The result of the in vivo test was because of the support of other organs; the result of the ex vivo test was because of the formalin fixation without support from other organs.

In addition to the whole liver in the original preseparation (Figure 4A), Rat B1’s discrete liver lobes were imaged (Figure 4B). The fused images of each liver lobe show dark spots in Lobes 1–4, and the red spot with the lowest intensity covered most of Lobe 5. Moreover, by using the same color scale, Figure 4B shows that the red on each separated liver lobe was more intense than that of all of the stacked liver lobes in Figure 4A. This occurred because each separated liver lobe on the same plane was at an identical distance from the scanning probe, whereas all of the liver lobes that were stacked together were at various distances from the probe. The lower lobes were the furthest from the scanning probe.

Moreover, a piece of the red spot region of the ex vivo images (Figure 4B) was stained using a hematoxylin and eosin (HE) stain and AFP stain. The morphology results of the HE staining revealed that most of the tissues in Lobes 1–4 exhibited abundant pink cytoplasms, whereas the entire region of Lobe 5 and a small portion of Lobes 3 and 4 exhibited purple because of normal amounts of cytoplasm. The 2 regions with light pink and dark purple in the HE stain corresponded to those with the dark brown and light brown in the AFP staining, respectively. Hence, these 2 regions were identified as tumor and normal tissues, respectively. Although small portions of normal tissue were present, the tissues were positive for tumors because of the presence of large tumor tissues. In other words, Lobes 1–4 were positive, but Lobe 5 was negative. Moreover, because the no-spot region of Lobes 1–4 in the ex vivo images yielded similar staining results to that of Lobe 5, this region was also negative. In summary, both the red-spot and no-spot regions in the ex vivo fused images of each lobe according to the dual-imaging model SSB correspond reasonably to the tissue staining results. The intensity of the red spot in the ex vivo images depended on the darkness of the AFP stain in the tumor tissue recognized by the HE stain.

Analytically, for each liver lobe of the 3 rats (Rats B1–3), its integral of I can be divided into levels higher and lower than 0.10 Volt.mm^2^ (Figure 4C). Similarly, its expression of the 2 tissue stains can determine positive and negative results. For example, for Rat B1 as shown in Figure 4B, positive results were confirmed by the expression of abundant pink cytoplasms in the HE stain and at least one brown region in the AFP stain in Lobes 1–4. The solid and dot pattern of the bar in Figure 4C represent the positive and negative results based on the 2 tissue stains. By comparing the integral of I and the judgment of positivity or negativity, a criterion was determined that the positive or negative judgment corresponds to the integral of I higher or lower than 0.10 Volt.mm^2^, respectively.

## Conclusion

This study developed a novel dual-imaging model SSB by integrating an optical camera and magnetic SSB to fuse low-field magnetic images of MNP distributions and optical images for simultaneous functional and structural imaging. The feasibility of this novel dual-imaging model SSB was verified by the favorable spatial agreement in phantom and animal tests, as well as confirmation from tissue staining. Hence, the application of imaging technologies of simple Fe_3_O_4_ MNPs were expanded from preoperative diagnosis by MRI to intraoperative positioning of tumors and preoperative imaging in clinics by using this novel dual-imaging model SSB, demonstrating the high potential of this method in the surgical navigation of MNP-targeted tumors in future clinical applications.

## Methods

### Synthesis and characteristics of anti-AFP reagent

The anti-AFP reagent used in this study was anti-AFP-meditated MNPs in a phosphate buffered saline solution. The magnetic core, surfactant, and bioprobe coating of anti-AFP-meditated MNPs were Fe_3_O_4_, dextran, and AFP antibody (EA502-Q1053, EastCoast Bio, USA), respectively. The basic materials of Fe_3_O_4_ MNPs solved in a water solution were obtained from MagQu Co., Taiwan, and the details of the synthesis process were described in a previous study [24].

The average hydrodynamic diameter of the anti-AFP reagent was 57.3 ± 15.2 nm, as measured using a nanoparticle size analyzer (Microtrac, Montgomeryville, PA, USA) [19]. The superparamagnetic property was observed in a magnetism-field curve obtained using a vibrating sample magnetometer (EG&G PARC, Newnan, GA).

### Development of a dual-imaging model of SSB

A dual-imaging model of SSB (Figure 1) composed of a scanning probe and a SQUID sensor unit was developed for optical and magnetic imaging, respectively. The proposed model differs from traditional SSB systems that measure magnetic signals alone. The unique design features of this scanning probe unit include a dual-imaging model mechanism, charge-coupled-device (CCD) module (Singapsy Enterprise Corp., Taiwan), and first-order vertical pickup coils surrounding the CCD module. The dual-imaging model mechanisms were inserted into the center of the circular excitation coil to construct the scanning probe unit. To measure the actual AC susceptibility of the MNPs, the excitation field strength and frequency of the excitation coil were set at 120 Oe and 400 Hz, respectively. The reasons for selecting this frequency included the low power loading at low frequencies for the same excitation coil, the interval between 360 and 420 Hz of low background noises [25], and the superior characteristics of AC-susceptibility technology. The field strength was selected to achieve sufficient sensitivity for few MNPs targeted in rats; this is discussed in the section describing the phantom test. Moreover, because the product of these 2 excitation parameters was substantially smaller than that required for animal biosafety [26], the excitation exhibited low risk.

The CCD module with a focus of 1 cm was not influenced by the magnetic fields, and it yielded an optical video with a resolution of 0.2 in per pixel and a wide angle of 40°. In general, the recorded video of each scanning line was automatically converted to a panoramic photograph. Subsequently, only the approximately central 2/3 region of the panoramic photograph was retained as a single line image, which was then combined with the other line images to construct a 2-dimensional optical image. Moreover, light-emitting diodes were arranged surrounding the CCD to provide adequate lighting during the scanning processes, which involved close distances between the scanning probe and sample. In addition, the 7-cm-diameter scanning probe unit can be operated similarly to an ultrasound probe with the assistance of a 3-dimensional step motor similar to that used in robotic scanners.

The dual-imaging model signals were acquired as follows. The optical video from the CCD module was recorded using a personal computer, although the magnetic signal was too low to be directly sensed by any current or voltage meter. The SQUID sensor unit was composed of a high critical-temperature T_c_ SQUID magnetometer (JSQ GmbH, Germany) in a dewar with a liquid nitrogen refrigerant and a set of shielding cans. Based on a typical conducting transfer coil [27] (as depicted in Figure 1), the weak magnetic flux was transferred from the pickup coils near the sample in an unshielded environment to the input coil surrounding the SQUID sensor, where it was amplified approximately 29-fold. The sensitivity of the entire dual-imaging model SSB was approximately 3 pT/√Hz. The white noise of the system is limited by the thermal noise of the normal-conducting flux transformer [27]. The environmental noise originated primarily from the concentric excitation coil and slightly from the CCD and cooling fan attached to the scanning probe unit.

The specificity of this dual-imaging model SSB in the measurement of anti-AFP reagents was characterized by conducting phantom and animal tests (Table 1).

**Table 1:** **Phantom and animal tests in this study**

**Test type**	**Test context**
**Phantom test**	**• Anti-AFP reagent:** 0.25 g in volume and 0.3, 0.2, 0.1, 0.01, 0.005 emu/g in concentration.
	**• Phantoms:** Micro test tubes were filled with anti-AFP reagent.
	**• Imaging:** The fused images composed of the magnetic image for the spatial distribution of anti-AFP MNPs and the optic image of micro test tubes.
**Animal tests**	**• Dose of Anti-AFP reagent:** 0.9 g in volume and 0.3 emu/g in concentration.
	**• Tumor rats:** Liver tumors were implanted on backs for 2 rats and in livers for 3 rats, separately.
	Anti-AFP reagents were intravenously injected through tail veins.
	**• Imaging:** The 2 tumor rats with back tumors were imaged in a prone position by using the dual-model SSB at 0 h.
	The 3 tumor rats implanted with liver tumors were imaged in a supine position at 0 h, 24 h, and with their bellies opened at 24.5 h.
	Both the stacked block and separated lobe livers of the 3 tumor rats implanted with liver tumors were magnetically examined ex vivo.
	**• Tissue stain:** After identifying the red-spot and no-spot regions in separated lobe livers by this dual-model SSB, small pieces of liver tissue in both regions for HE stain and AFP stain were performed.

### Phantom test

In the phantom test, microtest tubes (Eppendorf Corp., NY, USA) were filled with anti-AFP reagents at concentrations of 0.3, 0.2, 0.1, 0.01, and 0.005 emu/g. The samples weighed 0.25 g. Microtest tubes with a diameter of less than 8 mm were selected to imitate the size of early-stage tumors, and various concentrations of AFP reagent were used to simulate the dynamics of anti-AFP-meditated MNP-targeted tumors. In this test, the anti-AFP reagents of 0.01 and 0.005 emu/g were lower than the maximal MNP concentrations of approximately 0.045 emu/g in the livers of rats, as reported in the in vitro results of previous studies [22,23].

The anti-AFP reagents were scanned at a distance of 2–36 mm between the scanning probe and the top of the microtest tubes; this was approximately 31–65 mm between the scanning probe and the surface of anti-AFP reagents because the distance between the reagent surface and the top of the microtest tube was 29 mm. To calibrate the distance, 2 pieces of aluminum tape were attached to the upper surface of the samples and to the bottom surface of the scanning probe. Linking a copper wire from each aluminum tape to a resistance meter enabled the zero distance to be determined on the basis of the sound produced by the meter when the surface of aluminum tape contacted the wire. The relative position was controlled using the precision controller of a z-axis step motor. Each scanning path was commenced and terminated in free space, at least 1 cm from the animal body. The signal baseline of each line was determined according to the signal level in free space. Furthermore, the constant scanning speed and step size were 5 mm/s (for each line) and 5 mm, respectively. The pixel size of each line in the magnetic image was 5 mm, which was determined on the basis of the scanning path over the sampling points, and the line interval was 5 mm (Figure 2A). In other words, each pixel in the magnetic image was constructed according to the signal of a scanning step. The preliminary test showed that the measurement results of this scanning process were identical to those obtained using a static process. The remanence of the MNPs were omitted from the AC-susceptibility measurement.

### Animal test

Tumor masses of the GP7TB rat hepatoma cell line were implanted into 5 male F344/NNarl rats (age = 5 wk), which were divided into 2 groups. In one group, tumors were grafted onto the backs of 2 rats to enable easy identification of the tumors by using optical imaging; in the other group, the tumors were implanted in the livers of 3 rats to simulate real disease conditions. The tumors were confirmed using tissue stains. After 3 wk of incubation, anti-AFP reagents (dose = 0.9 g, concentration = 0.3 emu/g) were injected into the tail vein of these tumor rats. After administering a mixture of oxygen gas and isoflurane to anesthetize the rats, the tumor rats were imaged using the proposed dual-imaging model SSB. The imaging conditions were identical to those used in the phantom test. All experiments were conducted according to the animal care guidelines of National Taiwan University.

To verify the feasibility of tumor positioning by using the dual-model SSB, the tumor rats with back tumors were imaged in a prone position immediately before being injected with anti-AFP reagents (0 h), and again at 24 h after the injection (the tumor rat as shown in Figure 3). Each scanning time was approximately 2.5 minutes.

Similarly, to investigate the navigation of the interior liver tumors within the liver lobes, the 3 tumor rats with the implanted liver tumors were imaged in a supine position at 0 h, 24 h, and with their abdomens exposed at 24.5 h (Rat B1 as shown in Figure 4A). Each scanning time was approximately 8 minutes. The tumor rats were sacrificed and their livers were immersed in diluted formalin (concentration = 10%) for 1 wk. Both the stacked block and separated liver lobes were magnetically examined ex vivo (Rat B1 as shown in Figure 4B). Each scanning time was approximately 2 minutes. Opposite to the stacked livers, each liver lobe was observed at the same sample distance. After identifying the red-spot and no-spot regions in the separated liver lobes, small pieces of liver tissue in both regions were subjected to HE and AFP staining in the National Laboratory Animal Center, College of Medicine, at National Taiwan University.

## Abbreviations

MNPs: Magnetic nanoparticles
AFP: Antialpha-fetoprotein
SQUID: Superconducting-quantum-interference device
SSB: Scanning superconducting-quantum-interference-device biosusceptometry
CT: Computed tomography
MRI: Magnetic resonance imaging
PET: Positron emission tomography
AC: Alternate-current
CCD: Charge-coupled-device
LED: Light-emitting diode
T_c_: Critical-temperature
ACUC: Animal Care and Use Committee
HE: Hematoxylin-and-Eosin
